# Pelvic pain education – A short review on pelvic pain and endometriosis educational programs for adolescents

**DOI:** 10.1111/ajo.13856

**Published:** 2024-06-26

**Authors:** Hui Ping Adeline Thong, Amelia Kate Mardon, Susan Evans

**Affiliations:** ^1^ Faculty of Health and Medical Sciences University of Adelaide Adelaide South Australia Australia; ^2^ NICM Health Research Institute Western Sydney University Sydney New South Wales Australia

**Keywords:** adolescents, education, endometriosis, pelvic pain, review, school program

## Abstract

Persistent pelvic pain is a significant healthcare concern among adolescents; however adolescents often have poor health literacy regarding their pain. Current school curricula fail to specifically address pelvic pain and management strategies. This review aims to summarise current pelvic pain education programs in Australian and New Zealand schools. These programs have successfully strengthened the understanding of the psychosocial impact of periods and pelvic pain, instilled greater confidence in managing persistent pain and have allowed for prompt detection and treatment of pelvic pain in adolescents. An outcomes‐driven, collaborative, and coordinated approach is needed to improve pelvic health educational interventions for adolescents.

## INTRODUCTION

Persistent pelvic pain (PPP) is an umbrella term which encompasses a wide range of complex conditions associated with pain perceived in the pelvis for over six months.^1^ PPP conditions include recurrent distressing dysmenorrhoea, endometriosis, bladder pain syndrome, and inflammatory bowel disease, among others.^2^ PPP is highly prevalent among adolescents. Approximately 25% of Australian adolescents experience marked menstrual disturbance, including severe pelvic pain.^3^ In adolescents undergoing laparoscopy for pelvic pain, approximately 64% are diagnosed with endometriosis.^4^ Addressing PPP is crucial because of the significant impact on adolescents' psychosocial wellbeing; teens report increased anxiety and depression, poor school attendance, academic performance, and social withdrawal.^5^ Adolescents with PPP generally have poor menstrual and pelvic health literacy.^6^ Poor pelvic health literacy may be associated with higher levels of pain catastrophising and pain disability,^7^ as well as delayed medical attention due to normalisation of pain.^8^ Given the substantial burden of PPP, ensuring adolescents are knowledgeable about the condition is critical to promote early diagnosis and intervention.

Patient education is critical for improving clinical outcomes for adolescents with PPP. Pelvic health education is not mandated in the Australian and New Zealand school curricula; however, when it is discussed, the content predominately focuses on menstruation and ovulatory physiology. Education is often not provided about PPP or management strategies.^9^ The content taught is also highly dependent on the educator's knowledge. Certainly, teachers attribute poor health literacy on period pain in schools to their own poor mastery of the content, and lack of confidence in imparting such knowledge.^9^ Students across Australia expressed that existing measures to educate students about pelvic pain are inadequate.^9^ Students reported that education focused exclusively on biology and did not reflect common concerns they experience. Educational content was also taught too late, making it appear to be of lower utility value.^9^

School‐based programs provide a suitable platform for delivering timely, high‐quality education about PPP. School‐based ovulatory‐menstrual (OM) health literacy programs are centred around the physiology of menstruation. OM programs have been effective at improving functional health literacy, challenging misinformation, and helping adolescents become more familiar with their menstrual cycles.^10^ However, these programs do not discuss menstruation in the context of abnormal pelvic pain, or how to manage pain. Online education resources about self‐management techniques for PPP have increased engagement with outpatient gynaecology services by adolescents with PPP.^11^ Providing PPP education to adolescents may assist with timely diagnosis and potentially reduce the likelihood for recurrent episodes of severe dysmenorrhoea to predispose adolescents to prolonged periods of pain, via hyperalgesia priming,^12^ leading to other debilitating chronic pain conditions. Increased brain neuroplasticity in adolescence may contribute to the development of PPP, further emphasising the importance of ensuring early education for appropriate pain management.^13^ Providing PPP education programs to adolescents in schools will allow for a deeper understanding about PPP conditions, associated psychosocial implications, and appropriate management strategies.

## AIM

This narrative review summarises the current state of school‐based education programs about PPP and endometriosis for adolescents in Australia and New Zealand.

## MATERIALS AND METHODS

A search was conducted of the following databases (PubMed, Medline (OVID), Embase (OVID), Cochrane Library) from inception to 28 May 2023 with the search terms (‘persistent pelvic pain’, ‘endometriosis’, ‘health literacy’, ‘menstruation’, ‘adolescents’). The following keywords employed as part of the search were carefully considered; words that appeared frequently across known literature were selected as keywords for the literature search. Articles included were peer‐reviewed in academic journals, with preference given to systematic reviews and meta‐analyses, and English publications (Table 1). A grey literature search using Google Scholar was also performed, using search terms ‘persistent pelvic pain’ AND ‘adolescents’ AND ‘health literacy’. PPP literature was limited to adolescents (10–19 years).^14^ Program directors (individuals in charge of the programs which specialise in educating people about persistent pelvic pain in Australia and New Zealand) were contacted via email to gain further information on potentially relevant PPP education programs that do not have data published in academic journal articles (see Appendix S1).

**Table 1:** Search strategy of academic databases

	Search terms	Hits
Pubmed		
1	((persistent pelvic pain) AND (health literacy) AND (adolescents) OR (endometriosis)) NOT (menstruation)	181
2	((persistent pelvic pain) AND (health literacy) AND (adolescents) OR (endometriosis)) NOT (menstruation) NOT (period pain)	165
3	((persistent pelvic pain) or (chronic pelvic pain) AND (health literacy) AND (adolescents) OR (endometriosis)) NOT (menstruation)	181
4	((persistent pelvic pain) or (chronic pelvic pain) or (pelvic pain) AND (health literacy) OR (health education) AND (adolescents) OR (endometriosis)) NOT (menstruation)	559
Medline (OVID)		
1	‘Persistent pelvic pain’ OR ‘Pelvic pain’ or ‘Endometriosis’ AND ‘Adolescents’ .mp	382
2	‘Persistent pelvic pain’ OR ‘Pelvic pain’ or ‘Endometriosis’ AND ‘Adolescents’ NOT ‘Menstruation’ .mp	338
3	‘Persistent pelvic pain’ OR ‘Pelvic pain’ or ‘Endometriosis’ AND ‘Health literacy’ .mp	11
4	‘Persistent pelvic pain’ OR ‘Pelvic pain’ or ‘Endometriosis’ AND ‘Health literacy’ AND ‘Adolescents’ .mp	3
4	‘Persistent pelvic pain’ OR ‘Pelvic pain’ or ‘Endometriosis’ AND ‘Health literacy’ OR ‘Menstrual health’ .mp	361
Embase (OVID)		
1	‘Persistent Pelvic Pain’ OR ‘Period Pain’ OR ‘Endometriosis’ AND ‘Adolescent’ .mp [mp = title, abstract, heading word, drug trade name, original title, device manufacturer, device trade name, keyword heading word, floating subheading word, candidate term word]	1919
2	‘Persistent Pelvic Pain’ or ‘Period pain’ or ‘Endometriosis’ AND ‘Health Literacy’ AND ‘Adolescent’ .mp	3
3	‘Persistent Pelvic Pain’ OR ‘Period Pain’ AND ‘Health literacy’ AND ‘Adolescent’ .mp	1
4	‘Pelvic pain’ OR ‘Period Pain’ AND ‘Health Literacy’ AND ‘Adolescent’ .mp	2
*Cochrane*		
1	‘persistent pelvic pain’ OR ‘health literacy’ OR ‘adolescent’ OR ‘period pain’ OR ‘endometriosis’	214 914
2	‘persistent pelvic pain’ AND ‘health literacy’ OR ‘adolescent’ AND ‘endometriosis’	187
3	(persistent pelvic pain) AND (adolescents) AND (period pain) AND (health education) (Word variations have been searched)	6
4	(persistent pelvic pain) AND (adolescents) AND (period pain) AND (health education) (Word variations have been searched)	9
5	#2 or #3	205

## RESULTS

A summary of school‐based PPP educational programs in Australia and New Zealand is provided (Table 2).

**Table 2:** Summary of school‐based PPP educational programs in Australia and New Zealand

Study	Target population	Background/strategy	Implementers	Type of evaluation	Main results
Periods, Pain, Endometriosis Program Talk (PPEP Talk®)	Secondary school students across Australia assigned male at birth and students assigned female at birth	PPEP Talk® is rooted in contemporary pain science education and is aimed at clarifying misconceptions surrounding pelvic pain with a focus on the psychosocial impact of pelvic pain, identifying if the pain is normal and warrants further medical advice, understanding what endometriosis is and imparting them with constructive skills to manage pelvic pain	Pelvic Pain Foundation of Australia; supported by both federal and state governments in Australia through the National Action Plan for Endometriosis	Post‐program data audit	Post‐program audit data from PPEP Talk® demonstrated that 94% of people assigned female at birth found PPEP Talk® informative, 85% were empowered with new knowledge, and a reduction in people without information on endometriosis from 44% before PPEP Talk®, to 2% after PPEP Talk®
PeriodTalk™	9–13‐year‐old boys and girls	PeriodTalk™ provides education on premenstrual syndrome, pain management, sanitary products, and changes during puberty, over the course of four weeks, via educational videos and group activity simulations	Tash Lawton and Share the Dignity charity foundation	Post‐program data audit	PeriodTalk™ has rolled out to more than 100 schools in Australia, and has successfully increased levels of preparedness and confidence in dealing with menstrual pain and puberty, and helped to normalise conversations surrounding menstrual pain and destigmatising associated shame and embarrassment
Menstrual Health and Endometriosis (ME)	13–18‐year‐old students in private and public schools	Education on persistent pelvic pain, dysmenorrhoea and endometriosis is provided, with the aim of early detection of abnormal pelvic pain in mind	Educators from Endometriosis New Zealand (ENZ)	Evaluation by Bush and colleagues	There was an increase in the number of young women seeking specialist care after being involved in ME menstrual literacy programs

## PERIODS, PAIN, ENDOMETRIOSIS PROGRAM TALK (PPEP TALK®)

Periods, Pain, Endometriosis Program Talk (PPEP Talk®), is an initiative established by the Pelvic Pain Foundation of Australia and supported by both federal and state governments in Australia through the National Action Plan for Endometriosis. PPEP Talk® educates secondary school students across Australia about period pain, pelvic pain and endometriosis.^15^ PPEP Talk® helps adolescents understand whether their pain is normal, how the wide range of symptoms fit together, what endometriosis is, imparts them with skills to manage pelvic pain and teaches them when to seek medical advice. PPEP Talk® is also grounded in contemporary pain science. Traditional biological models of pain taught in schools faced criticism for suggesting a direct correlation of overall disease burden with levels of tissue injury.^16^ Interestingly, updated literature on pelvic pain and endometriosis demonstrates the extent of endometrial tissue on laparoscopic findings is inconsistent with overall disease burden and pain intensity.^17^ Providing students with contemporary pain science education is valuable in clarifying common misconceptions and helping students appreciate the multifaceted nature of pelvic pain, beyond tissue damage.^16^ Unlike traditional school curricula,^9^ PPEP Talk® discusses the psychosocial impact of pain to foster a more holistic understanding of the impact of PPP on lives. Students appear to benefit from attending PPEP Talk®; 94% of people assigned female at birth found PPEP Talk® informative, 85% felt empowered with new knowledge, and the proportion of people without information on endometriosis reduced from 44% before PPEP Talk®, to 2% after PPEP Talk®.^18^ PPEP Talk® also offers sessions to students assigned male at birth, to increase understanding about female pelvic pain, and help them to better support the women in their lives with pain.

## PeriodTalk™

Period Talk™ is a program which aims to educate boys and girls between the ages of nine and 13 years. Period Talk™ incorporates content on premenstrual syndrome, pain management, sanitary products, and changes during puberty, over the course of four weeks, via educational videos.^19^ Adolescents are taught how to use pads and tampons in group activity simulations, where blood is represented by red liquid. Adolescents are also educated about how premenstrual syndrome is a real normal experience, that can be variable between individuals. Such knowledge can help to destigmatise social stigma surrounding emotional reactivity, as part of premenstrual syndrome. The platform seeks to increase preparedness, normalise conversations on periods, and destigmatise associated shame and embarrassment. To date, Period Talk has been rolled out to over 100 schools.^19^ Such programs are beneficial in making knowledge surrounding menstruation and navigating puberty accessible to adolescents going through puberty, educators and parents alike, helping them to feel empowered, and puberty a less confronting experience.

## MENSTRUAL HEALTH AND ENDOMETRIOSIS

The menstrual health and endometriosis (ME) program involves teaching content on persistent pelvic pain, dysmenorrhoea and endometriosis, by educators from Endometriosis New Zealand (ENZ) to 13–18‐year‐olds in private and public schools. Bush et al conducted an assessment of ME education in New Zealand schools, affirming the benefit of such education and early detection of persistent pelvic pain.^20^ Bush and colleagues observed an increase in young women seeking specialist care after receiving menstrual literacy programs, affirming the success of ME.^20^ ME is also striving to develop an online platform to increase accessibility of the program.^20^

## CONCLUSION

Ultimately, efforts toward increasing pelvic pain literacy aim to foster a stronger understanding of pelvic pain. Programs such as PPEP Talk®, PeriodTalk™ and ME play a vital role in empowering adolescents to support themselves with pain education and pain management strategies and reducing the personal and societal burden of pelvic pain. PPEP Talk® has provided adolescents with a more holistic perspective of pelvic pain, with an emphasis on the physical and psychosocial impacts, and also providing practical pain management strategies. Similarly, PeriodTalk™ has been effective in reducing stigma surrounding premenstrual syndrome and misconceptions about menstrual pain, via educational videos and group activity simulations. The implementation of ME has seen an increase in early detection of pelvic pain and young women seeking specialist care via the provision of education on dysmenorrhoea, endometriosis and PPP. The integration of external school‐based programs appears successful in supplementing gaps in current school curriculum on pelvic health.

## Supporting information


**Appendix S1.** Additional contacts sourced for information.
