# Supplementary material for: Pelvic pain education – A short review on pelvic pain and endometriosis educational programs for adolescents
Source: Aust N Z J Obstet Gynaecol. 2024 Jun 26;65(1):25–9. doi: 10.1111/ajo.13856 (PMC11924169; doi:10.1111/ajo.13856)
Supplement: Supplementary file 1 — Appendix S1. Additional contacts sourced for information. [file AJO-65-25-s001.docx]

**Appendix 1**

**Additional contacts sourced for information:**

Contact 1
Jessica Taylor
President of QENDO
[jessica@qendo.org.au](about:blank)

Contact 2
Melissa Parker
Canberra Endometriosis Centre | Division of Women Youth and Children
[melissa.parker@act.gov.au](about:blank)

Contact 3

Tanya Cooke
Chief Executive of Endometriosis New Zealand
Email address [tanya@nzendo.org.nz](about:blank)
